# The Phosphorus Economy of Mediterranean Oak Saplings Under Global Change

**DOI:** 10.3389/fpls.2019.00405

**Published:** 2019-04-05

**Authors:** Inga Dirks, Julia Köhler, Shimon Rachmilevitch, Ina C. Meier

**Affiliations:** ^1^French Associates Institute for Agriculture and Biotechnology of Drylands, Ben Gurion University of the Negev, Beersheba, Israel; ^2^Plant Ecology, Albrecht-von-Haller Institute for Plant Sciences, University of Göttingen, Göttingen, Germany

**Keywords:** drought, eastern Mediterranean, global change, nitrogen deposition, phosphorus limitation, *Quercus calliprinos*, uptake efficiency

## Abstract

While a severe decrease in phosphorus (P) availability is already taking place in a large number of ecosystems, drought and nitrogen (N) deposition will likely further decrease the availability of P under global change. Plants have developed physiological strategies to cope with decreasing P resources, but it is unclear how these strategies respond to elevated N deposition and summer droughts. We investigated the influence of N and P availability and soil drought on P uptake (H_3_^33^PO_4_ feeding experiment) and use efficiencies in young *Quercus calliprinos* Webb. trees. We hypothesized that (H1) the expected increases in soil N:P ratios will increase the efficiencies of P uptake and use of oak saplings but will decrease the efficiencies of N uptake and use, whereas (H2) drought will affect P uptake efficiency more than N uptake efficiency. In confirmation of (H1) we found that a sharp increase of the soil N:P ratio from 4 to 42 g g^-1^ significantly increased the instantaneous ^33^P uptake efficiency (^33^PUptakeE) by five-fold and long-term P uptake efficiency (PUptakeE) by six-fold, while it decreased N uptake efficiency (NUptakeE) and N use efficiency (NUE). In contradiction to (H1), P use efficiency (PUE) did not respond to the simulated extended gradient of soil N:P ratios but remained relatively constant. (H2) was only partially confirmed as soil drought reduced PUptakeE by up to a fourth at high soil N:P ratios but had no significant effect on NUptakeE. As a consequence, increasing summer droughts may decrease the response of PUptakeE to increasing P limitation, which – in the absence of adjustments of the efficiency of P use – can aggravate growth reductions in this eastern Mediterranean tree species under global change.

## Introduction

Increasing N deposition and summer droughts under global change will induce nutritional imbalances and large-scale undersupply of essential plant nutrients such as P in a large number of natural forest ecosystems (Talkner et al., 2015; Sardans et al., 2016), but especially in Eastern Mediterranean forests where extreme climate conditions are already common and the effect of global change will be disproportional (Lelieveld et al., 2012). The deterioration of P availability is already indicated by decreasing P concentrations and increasing N:P ratios in leaves and fine roots, as well as by reports on growth reductions due to P limitations for various regions of Central and Southern Europe and elsewhere (Elser et al., 2007; Peñuelas et al., 2012). This trend of increasing P deficiency of forest trees is even more pronounced in regions where P availability had already been low before (Jonard et al., 2015): Mediterranean ecosystems are commonly characterized by geological substrates poor in mineral P content and by accumulation of occluded P with longer soil development (Singer, 2007; Cramer and Hoffman, 2015) and, thus, may shift from latent to apparent P deficiency under global change.

Plants can respond to P deficiency by increasing their P uptake and use efficiencies. P uptake is generally constrained since most P occurs sorbed to or occluded in Fe and Al oxides, precipitated as secondary mineral, or integrated in humic substances but not in plant-available form (Schachtman et al., 1998; Ragothama, 1999). In addition, the low diffusion coefficient of plant-available soluble H_2_PO_4_^-^ causes a distinct depletion zone in the rhizosphere. Accordingly, several adaptation or acclimation strategies for the increase of the rate and efficiency of P uptake exist. Among them the most fundamental plant response is to increase the root:shoot ratio, adjust the root architecture toward higher branching intensity, and increase root proliferation into unexplored soil regions (Lambers et al., 2006). In addition, plants can enhance the exudation of organic acids or the secretion of phosphatases and phytases, which increases the solubility of labile and organic P (Neumann and Römheld, 2007; Hofmann et al., 2016). Plant P uptake efficiency (PUptakeE) can also be increased directly by an elevated expression of high-affinity P_i_-transporters and other alterations of the membrane structure (Smith et al., 2000; Kavka and Polle, 2017) and by colonization with mycorrhizal fungi (Torres Aquino and Plassard, 2004; Cairney, 2011). In contrast to PUptakeE, increases in P use efficiency (PUE) are mostly achieved by internal reallocation of plant P to photosynthetic and growth functions (Lambers et al., 2012; Veneklaas et al., 2012).

Global change executes several constraints on the relation between P availability and PUptakeE: first, atmospheric N deposition from anthropogenic sources induces nutritional imbalances and accelerates the P demand of forest trees (Jonard et al., 2015). Second, the expected increases in the duration and intensity of summer droughts will likely further impair P availability due to decreased P mineralization (Schimel et al., 2007) and reduced mobility (Schachtman et al., 1998) and diffusion rates of P in drier soil (Kreuzwieser and Gessler, 2010). Secretion of phosphatases and expression of high affinity P_i_ transporters are additionally expected to be interfered by drought (Sardans and Peñuelas, 2007; Lang et al., 2016) as well as the mycorrhizal symbiosis (Kleczewski et al., 2010). These negative drought effects on P uptake seem to be even more pronounced than their effects on N uptake, leading to reductions in plant P storage (Sardans and Peñuelas, 2007) and further divergence of the N:P ratios with drought (He and Dijkstra, 2014).

In the dry eastern Mediterranean climate, Palestine oak (*Quercus calliprinos* Webb) is a common evergreen broadleaf tree species which is widely distributed from northern Algeria to southern Turkey and achieves predominance in the natural Mediterranean scrub woodland of Israel (Zohary, 1966; Schiller et al., 2003). Palestine oak grows on a relatively broad spectrum of soils and is used for reforestation, erosion control, and soil conservation. Yet despite its occurrence across a wide range of mean annual precipitation (from 460 to 1000 mm a^-1^ at higher elevation and altitude; Kutiel and Naveh, 1987; Klein et al., 2013), it is more drought sensitive than other co-occurring tree species and is limited by high temperatures (Helman et al., 2017) and low nutrient availability (Tognetti et al., 2000), which could reduce its growth and vitality under a future drier and warmer climate. The predicted climate change conditions in the eastern Mediterranean include faster warming than expected for the global mean, an increasing number of very hot days in summer, and a further decline in precipitation, especially in spring and summer (Lelieveld et al., 2012; Jacob et al., 2014). Concerns are rising that climate change in combination with N deposition will have disproportional effects on the P nutrition for the forest vegetation in this region compared to other areas – especially at sites that are currently already at the drought edge of distribution of Palestine oak.

To improve our understanding of the complex responses of P and N uptake and use efficiencies to the expected global change conditions, we exposed young Palestine oak trees to an increase in soil N:P ratios and a decrease in soil moisture in a two-factorial climate chamber experiment (3 N:P ratios × 2 soil moisture levels). We examined oak uptake and use efficiencies from a ^33^P feeding experiment, photosynthetic measurements, and elemental analyses of recent biomass. Subsequently, we analyzed the responses of P and N uptake and use efficiencies to decreasing P availability and decreasing soil moisture. We hypothesized that (H1) the increase in soil N:P ratios increases the efficiencies of P uptake and use of oak saplings but decreases the efficiencies of N uptake and use; and (H2) drought affects P uptake efficiency more than N uptake efficiency.

## Materials and Methods

### Plant Material and Experimental Set-Up

Our experiment was conducted with evergreen Palestine oak (*Quercus calliprinos* Webb.) saplings that were raised from seeds collected from a mature oak forest in the Lower Galilee in northern Israel (close to Turan; N 32° 46′, E 35° 22′; plant nursery: Keren Kayemeth LeIsrael, Jerusalem, Israel). In February 2015, 48 bare-rooted 1.5-year-old oak trees of similar size were carefully transported to the botanical garden in Göttingen, Central Germany, where they were rinsed with bi-distilled water and planted in 6-L pots filled with sterilized mineral sand / very fine gravel with a particle size of ≥2 mm, a soil texture that facilitates root studies. The potted oak saplings were placed in a randomized array in a greenhouse chamber with climate control (air temperature 21/18°C day/night, day length 12 h; in representation of the growing season conditions of Palestine oak between February and October) and were cultivated for 30 weeks until the start of the physiological and biochemical measurements in September 2015. The selected saplings had an average shoot height of 67 ± 2 cm (measured from the soil surface to the top of the sapling), a crown volume of 5130 ± 570 cm^3^, and a mean maximum stem diameter of 0.6 ± 0.1 cm. Oak saplings were re-randomized bi-weekly. We simulated a gradient of decreasing P availability in response to an increase in N deposition and decrease in soil moisture as expected under global change. We established two soil moisture conditions, a well-watered (M2; 10 % soil water content, v/v; close to field capacity) and a drought treatment (M1; 6.7% soil water content, v/v; close to the permanent wilting point) based on the texture of the sandy inorganic growth medium used in the experiment. Soil water contents were biweekly adjusted for plant water consumption by homogeneous irrigation with demineralized water to bring the soil back to its target soil moisture content. We simulated low (N1; 7.3 mg kg^-1^) and elevated N additions (N2; 15.2 mg kg^-1^), estimated on the basis of the extremely low N content of the sand used for the experiment (N_total_ = 0.14 g kg^-1^), typical soil N concentrations in the Mediterranean (0.8–4.9 g kg^-1^; Carreira et al., 1994; Evrendilek et al., 2006), and the expected N deposition rates in the eastern Mediterranean (15–22 kg N ha^-1^ yr^-1^; Roda et al., 2002; Christodoulaki et al., 2015). Our N addition schedule is, thus, a compromise between N deposition rates that are two to six times higher than the expected deposition rates (if extrapolated to a whole year: 44 and 91 kg N ha^-1^ yr^-1^) and final soil N concentrations that were still 5–33 times lower than their average in the eastern Mediterranean (final soil N: 0.15 and 0.16 g N kg^-1^). N was added weekly as KNO_3_, NaNO_3_, and NH_4_NO_3_ by watering the soil surface with a dissolved fertilizer. We did not consider leaf N uptake in our experiment. Finally, we established two P availability levels, a low (P2; 1.79 mg kg^-1^) and an extremely low P supply treatment (P1; 0.36 mg kg^-1^) on the basis of the estimated plant-available P content in the sandy substrate used for the experiment (*c.* 3 mg P_resin_ kg^-1^; *cf.* Köhler et al., 2018) and typical plant-available soil P concentrations in the eastern Mediterranean (4–11 mg P_bicarbonate_ kg^-1^; Henkin et al., 1994). P was added weekly as KH_2_PO_4_ by watering with a dissolved fertilizer. Our fertilization regime led to three N:P treatments: the relative N:P ratio increased from N1P2 (control; N/P = 4 g g^-1^) to N2P2 (elevated N addition; N/P = 8 g g^-1^) and N2P1 (elevated N addition and P limitation; N/P = 42 g g^-1^), with eight replicates per treatment. To avoid differences in salinity, we controlled for the electric conductivity of all stock solutions (σ ∼0.77 dS m^-1^). Micronutrients were added three times during the experiment with a diluted Hoagland solution.

### Photosynthetic Carbon Assimilation

Leaf gas exchange measurements were conducted at the end of September 2015 on one canopy leaf per sapling with an infrared CO_2_ analyzer (LI-6400; LI-COR Biosciences, Lincoln, NE, United States) during the midday hours, i.e., between 10.30 a.m. and 1.30 p.m. We measured area-based midday leaf photosynthesis (A_800_; μmol CO_2_ m^-2^ s^-1^) at high photosynthetically active radiation (800 μmol PAR m^-2^ s^-1^). During the measurements, average leaf temperature was 21°C, relative humidity 40%, vapor pressure deficit 12 hPa, and CO_2_ concentrations ambient (400 μmol CO_2_ mol^-1^ air). Mass-based leaf photosynthesis (in μmol CO_2_ g^-1^ s^-1^) was calculated from A_800_ and the ratio of leaf area per dry weight (specific leaf area, SLA; in cm^2^ g^-1^; for a description of the measurement see the description of the analysis of shoot biomass and morphology below). We calculated the instantaneous, late-summer photosynthetic P and N use efficiencies according to PPUE = A_800_^∗^SLA/P_Leaves_ and PNUE = A_800_^∗^SLA/N_Leaves_, respectively (in μmol CO_2_ g^-1^ P or N s^-1^), where *P_Leaves_* is the foliar P concentration and *N_Leaves_* the foliar N concentration (*cf.* Li et al., 2012; Gan et al., 2016).

### ^33^P Labeling Experiment

To measure ^33^P uptake capacity at the end of the experiment, half of the experimental saplings representing all treatments were selected for a H_3_^33^PO_4_ feeding experiment, which was conducted in the Laboratory for Radioisotope Research (LARI), University of Goettingen. Soils of the potted saplings were labeled with 100 mL H_3_^33^PO_4_ tracer solution (total activity per sapling 1 MBq ^33^P), which was evenly injected to the soil at ten injection points and three soil depths (2, 4, and 8 cm soil depth, respectively). After an application time of 120 min, saplings were immediately excavated and separated into fine roots (≤2 mm), coarse roots (>2 mm), the stem base (base to 10 cm stem height), the older top stem which had originated before the experiment (above 10 cm stem height), the younger top stem which developed during the experiment, the older leaves which had originated before the experiment, and the younger leaves which developed during the experiment. All compartments were immediately frozen in liquid N_2_ and stored at -80°C to avoid any further progressing of ^33^P. A subsample of mixed soil was frozen similarly and used for the determination of the specific ^33^P activity in soil (i.e., isotope dilution of ^33^P; determined from the amount of ^33^P per amount of soil inorganic P_i_; in Bq mg^-1^ P_i_; *cf.* Zavišić and Polle, 2018). All plant samples were placed for 7 days in a freeze drier (BETA I, Christ, Osterode/Harz, Germany), weighed, and ground (Mixer Mill MM 400; Retsch, Haan, Germany). Subsequently, ground samples were digested with 65% HNO_3_ at 180°C (*cf.* Heinrichs et al., 1986), filtered (filter paper grade MN 640 w; Macherey Nagel, Düren, Germany), and mixed with a scintillation cocktail (Rotiszint eco plus LSC cocktail; Roth, Karlsruhe, Germany). The ^33^P radioisotope signature was determined with a low activity liquid scintillation analyzer (Tri-Carb 3180 TR/SL; Perkin Elmar, Waltham, MA, United States). ^33^P uptake was determined separately for fine roots, coarse roots, and aboveground biomass. The mass-specific rate of ^33^P uptake (^33^PUptake) was calculated from the whole-plant amount of ^33^P after 2 h divided by the whole-plant biomass; and division of the quotient by the specific ^33^P activity (in g P g^-1^ DW d^-1^; *cf.* Zavišić and Polle, 2018). The instantaneous ^33^P uptake efficiency (^33^PUptakeE; in Bq kBq^-1^) was calculated according to ^33^PUptakeE = whole-plant ^33^P/soil ^33^P, where *whole-plant ^33^P* is the whole- plant amount of ^33^P and *soil ^33^P* is the amount of ^33^P in soil.

### Root and Shoot Biomass and Morphology

The remaining 24 plants were harvested during the following week and analyzed for biomass, morphology, and their N and P contents. Following the procedure of the ^33^P labeled saplings, plants were divided into their compartments, namely fine roots, coarse roots, stem bases, older top stems, younger top stems, older leaves, and younger leaves. Leaves and fine roots were dried for 24 h at 65°C until weight stability, while coarse roots and stems were dried for 72 h at 65°C until weight stability. Relative growth rates were calculated according to RGR = (ln DW_2_ - ln DW_1_)/(t_2_–t_1_), where *DW_1_* is the estimated dry weight at the beginning of the experiment in February 2015, *DW_2_* is the dry weight at the end of the experiment in September 2015, and *(t_2_–t_1_)* is the length of the experiment (210 days). Biomass at the beginning of the experiment was estimated from the stem diameter measured at the beginning of the experiment and the relationship between dry weight and stem diameter *D* established at the end of the experiment from tree harvests (DW = 8.25 ^∗^ D – 11.46; *R^2^* = 0.52; in g). Twenty leaves per sapling were scanned to determine leaf area by using a flat-bed scanner and the computer program WinFOLIA (version 2014c; Régent Instruments Inc., Canada) and their dry mass was determined. Based on this information, SLA (in cm^2^ g^-1^) was calculated. Total leaf area was extrapolated based on the subsamples.

### P and N Uptake and Use Efficiencies

Dry plant compartments were ground with a vibratory disk mill (Scheibenschwingmühle TS; Siebtechnik GmbH, Mülheim, Germany) and subjected to high-pressure chemical digestion with 65% HNO_3_ at 195°C. The digested samples were analyzed for P by an inductively coupled plasma optical emission spectroscopy (ICP-OES, Optima 5300 DV; Perkin Elmer, Germany). Total N in the dried plant compartments were analyzed with an elemental analyzer (vario EL III; elementar, Langenselbold, Germany). We calculated the long-term P uptake efficiency (PUptakeE; in g P g^-1^ P_added_) by dividing the content of P in recent biomass, i.e., in fine roots and leaves that developed during the experiment, by its amount added by fertilization during the 2015 growing season. P use efficiency (PUE; in g DW g^-1^ P_DW_) was calculated from the increment in total biomass (dry weight) for a given increase in P in biomass during a given time period (*cf.* Koide, 1991). Correspondingly, N uptake efficiency (NUptakeE) and N use efficiency (NUE) were calculated by considering N instead of P.

### Data Processing and Analysis

Statistical analyses were performed with R, version 3.5.1 (R Development Core Team). Significance was determined at *p* ≤ 0.05. Means and standard errors were calculated from eight replicates per treatment for P, and four replicates for N. All datasets were log_10_ transformed to resemble normality and homogeneity of residuals. The probability of fit to normal distribution was tested by a Shapiro-Wilk test. We analyzed the effects of the soil N:P ratio and soil moisture on the analyzed parameters in two-way analyses of variance (ANOVA), where both effects were treated as fixed effect. Tukey’s range *post hoc* tests (Tukey’s honest significant difference test) were conducted to identify significant differences between individual factor levels. We calculated linear regressions to identify relationships between variables.

## Results

### Plant P Concentrations and N:P Ratios in Recent Biomass

**FIGURE 1:**
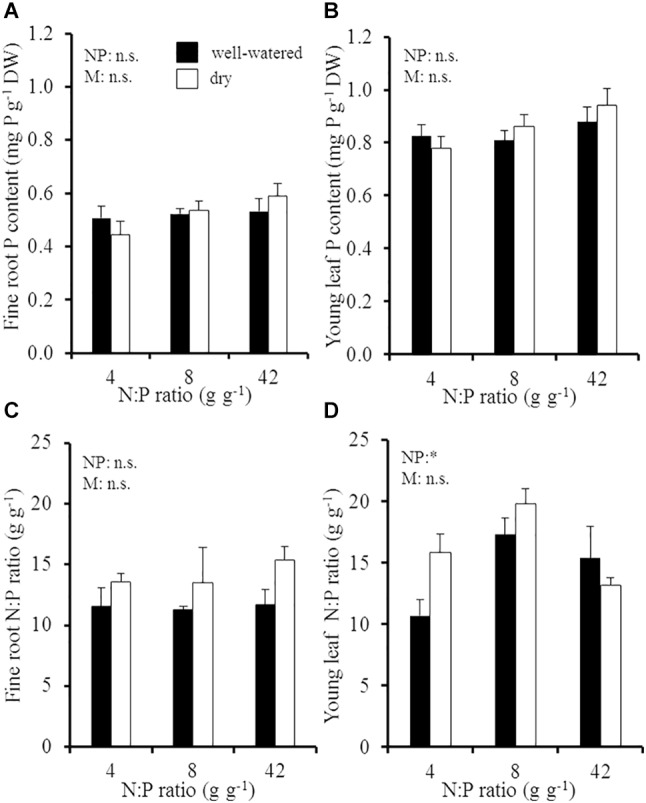
**(A)** Fine root P concentration, **(B)** young leaf P concentration, **(C)** fine root N:P ratio, and **(D)** young leaf N:P ratio of young Palestine oak (*Quercus calliprinos*) trees grown at increasing soil N:P ratio and decreasing soil moisture in a climate chamber. Shown are means and standard errors for replicate saplings (*n* = 48 for P concentrations; *n* = 24 for N:P ratios). The results of two-way ANOVAs on the significance of the effect of the soil N:P ratio (NP) or soil moisture (M) are indicated by asterisks (^∗^ for *p* ≤ 0.05; n.s., not significant).

Despite the extended soil N:P gradient and different soil moisture conditions, P concentrations in fine roots and young leaves remained relatively constant in different environmental conditions (Figure 1). Fine roots had on average a P concentration of 0.5 ± 0.02 mg P g^-1^ DW, while the average foliar P concentration in young leaves was slightly higher at 0.9 ± 0.02 mg P g^-1^ DW. The corresponding N:P ratios in fine roots responded to soil moisture conditions and were tendentiously higher in dry than in well-watered soil (increase from 13.5 g g^-1^ in moist soil to 15.3 g g^-1^ in dry soil; *p* = 0.051; Figure 1C), which resulted from a relative increase in fine root N concentrations in dry soil and not from a change in fine root P concentrations. In contrast to foliar P concentrations, N:P ratios in young leaves were significantly influenced by the soil N:P ratio (Figure 1D and Supplementary Table 1). The foliar N:P ratio increased from 11 ± 1 g g^-1^ at the lowest soil N:P ratio to 15–17 g g^-1^ at intermediate and high soil N:P ratios in well-watered soil, since foliar N concentrations increased at higher soil N:P ratios in well-watered soil. In dry soil, the foliar N:P ratio increased from 16 ± 1.5 g g^-1^ to 20 ± 0.5 g g^-1^ from the lowest to the intermediate soil N:P ratio; but it decreased to 13 ± 0.6 g g^-1^ at the highest soil N:P ratio (*p* > 0.05).

### Plant Biomass and Photosynthetic Carbon Assimilation

Over the course of the experiment, oak biomass was not influenced by the soil N:P ratio but was significantly reduced by soil drought (on average -18%; Supplementary Figure 1). The reduction of plant biomass in dry soil was the most distinct at the highest soil N:P ratio, at which plant biomass was reduced by more than a third. This decrease of plant biomass occurred mostly for old leaves (because of premature litterfall), fine root biomass, and coarse root biomass, while the decrease of young leaf biomass was less pronounced.

**FIGURE 2:**
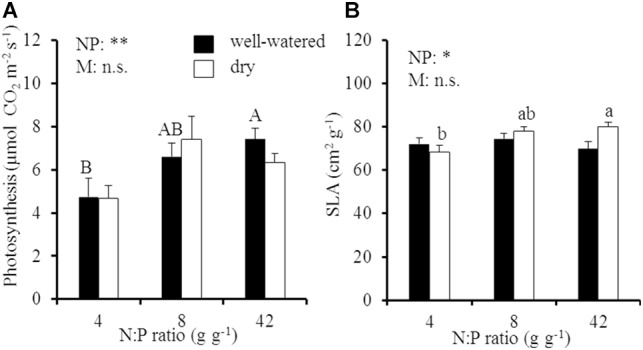
**(A)** Area-based instantaneous late-summer photosynthesis (A_800_) and **(B)** specific leaf area (SLA) of young Palestine oak (*Quercus calliprinos*) trees grown at increasing soil N:P ratio and decreasing soil moisture in a climate chamber. Shown are means and standard errors for replicate saplings (*n* = 24). The results of two-way ANOVAs on the significance of the effect of soil N:P ratio (NP) or soil moisture (M) are indicated by asterisks (^∗^ and ^∗∗^ for *p* ≤ 0.01 and 0.05; n.s., not significant). Significant differences at *p* ≤ 0.05 between soil N:P ratios are indicated for well-watered soil by different upper-case letters and for dry soil by different lower-case letters.

By contrast to oak biomass, leaf morphology and photosynthesis (A_800_) responded to a change in the soil N:P ratio but were not influenced by soil drought (SLA, *p* = 0.34 for the soil moisture effect; A_800_, *p* = 0.89 for the soil moisture effect; Figure 2 and Supplementary Table 1). Area-based carbon (C) assimilation of oak leaves increased from 4.7 to 7.4 μmol CO_2_ m^-2^ s^-1^ in well-watered soil (*p* < 0.05 for the N:P effect) and from 4.7 to 6.4 μmol CO_2_ m^-2^ s^-1^ in dry soil (*p* > 0.05 for the N:P effect), respectively, with an increase of the soil N:P ratio from 4 to 42 g g^-1^ (Figure 2A). Meanwhile, SLA significantly increased from 68 to 80 cm^2^ g^-1^ with an increase of the soil N:P ratio in dry soil (Figure 2B), which was also accompanied by an increase of mass-based photosynthesis at high N:P ratios in dry soil (Supplementary Figure 2).

### P and N Uptake Efficiencies in Response to Soil N:P Ratios and Drought

**FIGURE 3:**
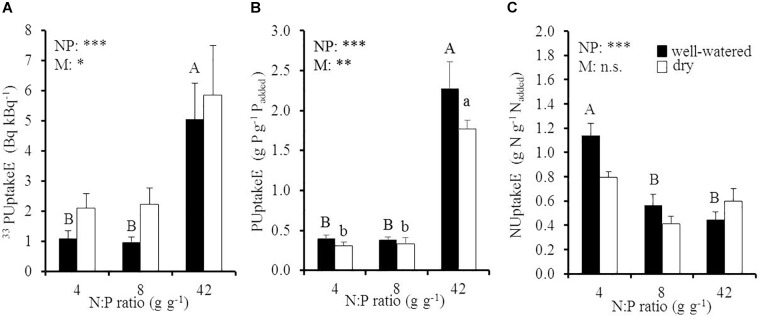
**(A)** Instantaneous ^33^P uptake efficiency (^33^PUptakeE), **(B)** long-term P uptake efficiency (PUptakeE), and **(C)** N uptake efficiency (NUptakeE) of young Palestine oak (*Quercus calliprinos*) trees grown at increasing soil N:P ratio and decreasing soil moisture in a climate chamber. Shown are means and standard errors for replicate saplings (*n* = 48 for PUptakeE; *n* = 24 for ^33^PUptakeE and NUptakeE). The results of two-way ANOVAs on the significance of the effect of the soil N:P ratio (NP) or soil moisture (M) are indicated by asterisks (^∗∗∗^, ^∗∗^, and ^∗^ for *p* ≤ 0.001, 0.01, and 0.05; n.s., not significant). Significant differences at *p* ≤ 0.05 between soil N:P ratios are indicated for well-watered soil by different upper-case letters and for dry soil by different lower-case letters.

Elevated soil N:P ratios significantly increased instantaneous ^33^PUptakeE and long-term PUptakeE and decreased NUptakeE (Figure 3 and Table 1). More specific, instantaneous ^33^PUptakeE increased by 4.6 and by 2.8 times under well-watered and dry conditions, respectively, when the soil N:P ratio increased by 5.3 times from 8 to 42 g g^-1^ (Figure 3A). The majority of the ^33^P was retrieved from fine and coarse root biomass and only a fraction from aboveground biomass, independent of the respective treatment (Supplementary Figure 3). Long-term PUptakeE increased by six times under well-watered and dry conditions, respectively, when the soil N:P ratio was raised by five times, i.e., when relative P availability was reduced (Figure 3B). Despite the influence of the factors soil N:P ratio and soil moisture on instantaneous ^33^PUptakeE and long-term PUptakeE, they had no cross-effect (*p* > 0.87; Table 1). We found no effect of the fine root:leaf biomass ratio on PUptakeE or NUptakeE in moist soil (PUptakeE: *p* = 0.39; NUptakeE: *p* = 0.81; not shown). Yet the absolute phosphorus uptake into recent biomass (young leaves and fine roots) related to the young leaf:fine root biomass ratio in dry soil (*R*^2^ = 0.58, *p* < 0.001), where P uptake rates increased by 17 μg P d^-1^ with an increase of the young leaf:fine root biomass ratio by 1 g g^-1^ (not shown). PUptakeE of oaks increased with fine root biomass (*R* = 0.56, *p* = 0.01) and RGR (*R* = 0.57, *p* < 0.001) in well-watered soil, but decreased with increasing amount of old leaf biomass (*R* = -0.50, *p* = 0.02) in dry soil.

**Table 1:** Two-factorial analyses of variance (ANOVA) on the significance of the effects of the soil N:P ratio (NP), soil moisture (M), and their interaction on the variance of instantaneous ^33^PUptake efficiency (^33^PUptakeE), long-term P uptake efficiency (PUptakeE), P use efficiency (PUE), instantaneous late-summer photosynthetic P use efficiency (PPUE), N uptake efficiency (NUptakeE), N use efficiency (NUE), and instantaneous late-summer photosynthetic N use efficiency (PNUE) of young Palestine oak (*Quercus calliprinos*) trees.

	^33^PUptakeE		PUptakeE		PUE		PPUE	
				
	*F*	*p*	*F*	*p*	*F*	*p*	*F*	*p*
NP	**13**.**3**	<**0**.**001**	**115**.**4**	<**0**.**001**	1.4	0.25	**5**.**6**	**0**.**01**
M	**5**.**0**	**0**.**04**	**4**.**7**	**0**.**002**	1.0	0.32	0.0	0.94
NP^∗^M	0.1	0.87	0.3	0.97	0.31	0.74	0.5	0.58

			**NUptakeE**		**NUE**		**PNUE**	
					
			***F***	***p***	***F***	***p***	***F***	***p***

NP			**14**.**3**	<**0**.**001**	**4**.**9**	**0**.**03**	2.3	0.14
M			1.6	0.20	**4**.**9**	**0**.**05**	0.3	0.61
NP^∗^M			2.7	0.11	0.2	0.79	1.5	0.27


*Given are *F* values and the probabilities of error *p*. Response variables were log-transformed to resemble normality. Significant effects (*p* ≤ 0.05) are indicated by bold letters (*n* = 24 for ^*33*^PUptakeE, NUptakeE, NUE, and PNUE; *n* = 48 for PUptakeE, PUE, and PPUE).*

The reduction in NUptakeE with an increase in soil N:P ratio was far less pronounced than the concurrent increase in PUptakeE: NUptakeE was reduced by half in well-watered conditions when the soil N:P ratio increased from 4 to 8 g g^-1^, i.e., when the relative N availability doubled, but it did not decrease much further at higher soil N:P ratio. It must be noted that both instantaneous ^33^PUptakeE and long-term PUptakeE were drought-sensitive, while NUptakeE was not influenced by soil moisture (*p* = 0.20; Table 1). According to our regression analyses, the uptake efficiencies for P and N did not relate (Supplementary Figure 4A).

### P and N Use Efficiencies in Response to Soil N:P Ratios and Drought

**FIGURE 4:**
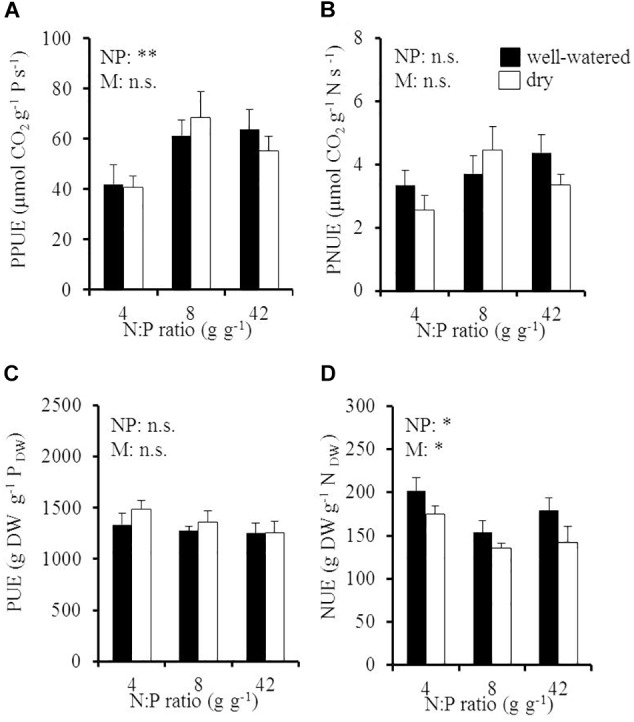
**(A)** Instantaneous late-summer photosynthetic P use efficiency (PPUE), **(B)** instantaneous late-summer photosynthetic N use efficiency (PNUE), **(C)** P use efficiency (PUE), and **(D)** N use efficiency (NUE) of young Palestine oak (*Quercus calliprinos*) trees grown at increasing soil N:P ratio and decreasing soil moisture in a climate chamber. Shown are means and standard errors for replicate saplings (*n* = 48 for PPUE and PUE; *n* = 24 for PNUE and NUE). The results of two-way ANOVAs on the significance of the effect of the soil N:P ratio (NP) or soil moisture (M) are indicated by asterisks (^∗∗^ and ^∗^ for *p* ≤ 0.01 and 0.05; n.s., not significant).

Elevated soil N:P ratios significantly increased instantaneous, late-summer PPUE from 41 to 65 μmol CO_2_ g^-1^ P s^-1^ with an increase of the soil N:P ratio from 4 to 8 g g^-1^, while soil moisture had no significant effect (Figure 4A and Table 1). PPUE significantly increased with foliar N concentration in young leaves (Supplementary Figure 5A) and with increasing instantaneous, late-summer PNUE (Supplementary Figure 5B) in both soil moisture treatments. PUE was on average 1328 ± 39 g DW g^-1^ P_DW_ and was neither influenced by soil N:P ratio nor by soil moisture (Figure 4C). PUE significantly increased with NUE in well-watered soil (*R*^2^ = 0.69; Supplementary Figure 4B) but was independent from PUptakeE (Supplementary Figure 4C).

N use efficiencies responded in antagonism to the P use efficiencies to environmental conditions. Late-summer PNUE was not influenced by the soil N:P ratio or soil moisture (*p* = 0.14 for the N:P effect; *p* = 0.54 for the soil moisture effect; Figure 4B), whereas NUE responded to both the soil N:P ratio and the moisture content (Figure 4D). Accordingly, NUE significantly decreased from 201 to 153 g DW g^-1^ N_DW_ with a doubling of the soil N:P ratio in well-watered soil. Drought further reduced NUE to 142 ± 19 g DW g^-1^ N_DW_ at high soil N:P ratios (significant decrease by -18%). NUE increased with NUptakeE in dry soil (*R*^2^ = 0.54, *p* ≤ 0.05; Supplementary Figure 4D).

## Discussion

Elevated N deposition and summer droughts in the course of global change are assumed to deteriorate the availability of P for important forest trees, but it remains unclear to what extent plants may adapt their uptake and use efficiencies for P and N under these conditions. Here we show that both short and long-term PUptakeE and late-summer PPUE increased with increasing soil N:P ratio, whereas PUE did not change. At the same time, both NUptakeE and NUE decreased with increasing soil N:P ratio, that is, with increasing N availability. Drought diminished the response of PUptakeE to increasing P limitation and decreased growth but had no significant effect on late-summer PPUE, PUE, and long-term NUptakeE. It seems that the efficiency of P use can be less responsive to the soil N:P and soil drought than the efficiency of P uptake in this Mediterranean oak species.

### Acclimation of P Acquisition to Increasing Soil N:P Ratios

Our study demonstrates that Palestine oak can adjust its capacity for P acquisition when soil P is the limiting element to the extent that P-limitation of PUE and growth do not occur. At the same time, they down-regulate the uptake efficiency for non-limiting soil N, which is probably saving energy for the plant. In nutrient-limiting soil one of the most effective plant strategies to increase nutrient acquisition is increased resource allocation to the root system. According to optimal partitioning theory, plants allocate carbohydrates and nutrients preferentially to those organs that acquire the most growth-limiting resource, with the consequence of growth being equally limited by all resources (Bloom et al., 1985; Friedlingstein et al., 1999). Accordingly, optimal partitioning theory predicts that relative carbon allocation to fine roots and the fine root:leaf biomass ratio will increase with a limitation of soil P, but decrease with an increase in N supply. We found no effect of the fine root:leaf biomass ratio on either PUptakeE or NUptakeE, probably a consequence of the opposed influences by decreasing P supply at increasing N supply in our study. Other authors demonstrated that higher capacity for nutrient uptake and assimilation related positively to absolute root and net primary production (Norby and Iversen, 2006) and, thus, to RGR of the investigated deciduous tree species (Gan et al., 2016). Similarly, we also found a positive relationship of PUptakeE with fine root biomass and RGR in the investigated evergreen oaks. Yet this relationship is probably not the cause but rather the consequence of a more efficient P uptake, which may have been induced at the morphological (e.g., greater fine root surface area), physiological (e.g., enhanced exudation of exoenzymes involved in the decomposition of organic P), or even cellular level (e.g., increased mRNA levels of genes involved in P and N absorption and metabolism; Gan et al., 2016). While we can exclude root morphology as well as organic P decomposition as significant factors for PUptakeE of oak in our pot experiment, we can only speculate if changes at the cellular level, i.e., in the membrane structure and in the density and affinity of P_i_-transporters, were the reason for increased PUptakeE of oaks under P limitation. This question of the effect of changes at the cellular level on PUptakeE of evergreen tree species deserves further study.

In contrast to our initial hypothesis, Palestine oak adjusted the efficiency of P uptake, but not the efficiency of P use under P limitation. Among different plant species, acclimation strategies to nutrient limitation differ profoundly: some species respond with changes in nutrient uptake efficiency, others with changes in nutrient use efficiency, and still others with changes in both (Nitschke et al., 2017). By contrast, PUE increased exponentially and at a higher rate than PUptakeE with increasing soil N:P ratio in temperate European beech (Köhler et al., 2018). Differences in PUE can be caused by either changes in plant P concentrations at constant biomass (Veneklaas et al., 2012; Gan et al., 2016) or changes in biomass production at constant plant P concentrations (Gan et al., 2016; Zavišić and Polle, 2018; Zavišić et al., 2018). The pronounced increase in PUE in deciduous European beech was mainly caused by a decrease in plant P concentration with increasing soil N:P, while the evergreen oak saplings of the current study had throughout a range of soil N:P ratios a comparably constant P concentration in plant tissues and constant biomass production. It seems that the young oaks have a P use economy which is independent from soil P availability. In times of low P availability, P from storage in vacuoles of coarse roots (Zavišić et al., 2018) can make plants autonomous from the amount of soil P, but this will consequently increase PUE (Gan et al., 2016). Accordingly, prominent use of P from storage in P-limited Palestine oak seems unlikely. Whereas decreased NUE under P deprivation was probably due to less efficient, waste use of N and reduced N storage in bark and wood when P (and not N) was limiting growth. NUE of the investigated Palestine oaks (135–201 g DW g^-1^ N_DW_) was even much lower than NUE of other evergreen oak species (578–1469 g DW g^-1^ N_DW_; Silla and Escudero, 2004). It appears that Palestine oaks can sufficiently adjust their PUptakeE to soil P availability to circumvent a shift in their PUE, while they are more opportunistic with respect to NUE.

When comparing European beech and Palestine oak it appears that PUE of the evergreen Mediterranean oak saplings (1249–1487 g DW g^-1^ P_DW_; current study) was much less variable and in the mid-range of the PUE of the deciduous temperate beech saplings (436–2260 g DW g^-1^ P_DW_; Köhler et al., 2018). While it has often been assumed that the dominance of evergreens in their low-nutrient habitats is due to higher nutrient use efficiencies than in deciduous species, comparisons at the whole plant level show that this is not the case (Aerts, 1995). Dominant plants on nutrient-poor and seasonally dry Mediterranean soils generally tend to have lower tissue N and P concentration in their evergreen leaves, which have longer lifespan and lower nutrient loss rates than deciduous leaves (Ribet and Drevon, 1996; Aerts and Chapin III, 2000), but they do not necessarily use foliar nutrients more efficiently for plant growth. This was also the case in the evergreen oaks, which had foliar P concentration at the lower range of the foliar P concentrations of deciduous beech from previous studies (oak: 0.8–0.9 mg P g^-1^ DW; beech: 0.5–2.8 mg P g^-1^ DW; Köhler et al., 2018; Zavišić et al., 2018). Yet, foliar P concentration also decreases with increasing plant size (Elser et al., 2010), which may also have caused lower plant P concentrations in the three times taller Palestine oak saplings of the current study than in the much smaller European beech saplings of the previous study (Köhler et al., 2018).

### Stoichiometric Homeostasis and Photosynthetic P Use Efficiency

Foliar and shoot P concentrations of Palestine oak were surprisingly constant at 0.8 mg P g^-1^ DW across the wide range of soil N:P ratios of our greenhouse study. Other field studies also showed that foliar P concentrations do not reflect soil P availability across extended P gradients (Bauer et al., 1997; Talkner et al., 2015; Lang et al., 2016), since leaves were preferred allocation sinks for P under P-poor conditions, which kept foliar P concentrations constant (Zavišić et al., 2018). This phenomenon is referred to as homeostasis, i.e., the degree to which an organism maintains its nutrient concentration around a given species-specific and size-specific value, despite variation in the availability of soil resources (Elser and Hamilton, 2007; Wang et al., 2012). Foliar P homeostasis in Palestine oak was probably maintained by acclimation of P acquisition and plant growth, with the latter having a stronger effect on foliar P homeostasis than on foliar N homeostasis. It is known that foliar P homeostasis differs from N homeostasis, since foliar P concentrations decrease more rapidly with plant size than N concentrations, resulting in a ^2^/_3_ slope for the N:P scaling relationship among different plant species (Reich et al., 2010). Accordingly, foliar N:P ratios increase systematically with decreasing leaf nutrient concentrations in larger plants, which represents a disproportionate investment in N-rich proteins for high photosynthetic rates and a rising return (*sensu* Niklas et al., 2007). This allometric scaling of N and P in plants is consequence of the dependence of both acquisition and use efficiency of foliar P on the acquisition and use of N and *vice versa* (biochemically dependent co-limitation hypothesis; Ågren et al., 2012). Yet stoichiometric homeostasis is not only dependent on plant size, but also on environmental conditions, which have been suggested to exert stronger control on foliar P concentrations than on foliar N concentrations (Elser et al., 2010). In contradiction, we observed foliar P concentrations in Palestine oak saplings that remained relatively constant across different environmental conditions (and probably mainly depended on sapling size), while foliar N concentrations varied as a function of soil N availability. This led to higher foliar N:P ratios and enhanced relative P limitation when soil N availability was increased. This increase in foliar N:P ratios in Palestine oak saplings observed in our study (11–17 g g^-1^) is comparable to other studies investigating trees in P-limited environments (8–24 g g^-1^; Dijkstra et al., 2016).

The constant foliar P concentrations across a range of soil N:P ratios probably maintain photosynthetic rates of Palestine oak. In general, foliar P concentrations directly correlate with photosynthesis across various plant species (Wright et al., 2004a; Hidaka and Kitayama, 2009), where decreased foliar P concentrations often decrease photosynthetic rates (Pieters et al., 2001). P limitation was shown to restrict photosynthesis and leaf dark respiration in different climatic regions (Kattge et al., 2009; Domingues et al., 2010). Accordingly, Palestine oak may circumvent P limitation of photosynthesis by homeostasis of foliar P concentration even at the most extreme soil N:P ratios. Even more, late-summer photosynthesis and PPUE of oak even increased with P limitation. Woody plants can increase PPUE at constant foliar P concentration by allocating more P to the metabolites that participate in photosynthesis and less P to compounds such as nucleic acids and phospholipids, which are not involved directly in C fixation (Lambers et al., 2012). In addition, increases in SLA at high soil N:P ratios as in our study relate to higher levels of mass-based photosynthesis (Supplementary Figure 2; Hidaka and Kitayama, 2009), even when foliar biomass is constant. Both a change in leaf morphology and an allocation shift to photosynthetic active metabolites can be reason for increases in late-summer PPUE and increases in photosynthetic C gain of Palestine oak under enhanced P limitation. Notwithstanding this increase in late-summer PPUE, it was still comparably low in this evergreen tree species in global comparisons (Palestine oak: 55.4 ± 3.3 μmol CO_2_ g^-1^ P s^-1^; global average: 103 μmol CO_2_ g^-1^ P s^-1^; Wright et al., 2004b; Proteaceae: 305 μmol CO_2_ g^-1^ P s^-1^; Lambers et al., 2012).

### Dependence of the P Economy on Soil Drought

Soil drought reduced the capacity of Palestine oaks to adjust their P acquisition to the availability of soil P. The adjustment of PUptakeE to low soil P decreased with increasing amount of old leaf biomass. In dry soil, evergreen oaks reduced their old leaf biomass at high soil N:P, probably by premature leaf drop, while sustaining a similar young leaf biomass. This decrease in leaf surface area of metabolically less active old leaves at high soil N:P ratio probably pre-empts dehydration and is primarily a drought stress syndrome characteristic for Mediterranean trees (Chaves and Oliveira, 2004). Premature leaf drop can enhance nutrient transport via sap flow, when it is improving the water status of the plant, but it also comes at a cost, since it comprises shorter P residence time in old leaves and canopy P losses, thus decreases PUE (Gleason et al., 2009). In the investigated Palestine oaks, premature leaf drop was not enough to circumvent a decrease in PUptakeE at high soil N:P ratio in dry soil. This decrease in PUptakeE was likely mainly a physical consequence of decreased mobility (Schachtman et al., 1998) and diffusion rates (Kreuzwieser and Gessler, 2010) of P in dry soil. PUptakeE was more strongly affected by drought than NUptakeE, which leads to further divergence of the foliar N:P ratios at high soil N:P ratio in dry soil (He and Dijkstra, 2014). As a consequence, growth of Mediterranean trees is increasingly P-limited in dry soil, unless PUE increases from the enhanced use of stored P from the vacuoles (Sardans and Peñuelas, 2007). The young oak trees of our study maintained their foliar P concentrations and PUE despite the P loss from premature leaf drop in dry soil constant, probably because of nutrient conservation from P resorption from senescent leaves and decreased tree biomass and growth. In global comparisons, foliar P concentrations and PUE of different plant species tend to be more strongly associated with mean annual precipitation than foliar N concentrations and NUE (Elser et al., 2010). This contrasts with our single-species study with Mediterranean oak, which is adapted to summer droughts and low soil P availability and mainly experiences disturbance of the efficiency of N use by soil drought. This decrease in NUE when soil moisture is limiting plant growth points at a less efficient, waste use of N under these circumstances.

## Conclusion

We investigated the effects of global change conditions on the P and N uptake and use efficiencies of evergreen Palestine oak trees, which are representatives of the Eastern Mediterranean scrub woodland. Our results demonstrate that increasing summer droughts will likely intensify nutrient imbalances for oak growth. Summer droughts decrease the acclimation of PUptakeE to increasing soil P limitation under elevated N deposition, leading to rising N:P imbalances in plants. Due to the absence of adjustment in the efficiency of P use in the investigated Palestine oaks, this effect may aggravate growth reductions from both water and P limitation under global change.

## Author Contributions

IM conceived and designed the research project. ID and JK performed the research. ID and JK analyzed the data. IM and ID wrote the manuscript. All authors approved the final version of the manuscript.

## Conflict of Interest Statement

The authors declare that the research was conducted in the absence of any commercial or financial relationships that could be construed as a potential conflict of interest.
