# Supplementary material for: The Phosphorus Economy of Mediterranean Oak Saplings Under Global Change
Source: Front Plant Sci. 2019 Apr 5;10:405. doi: 10.3389/fpls.2019.00405 (PMC6459984; doi:10.3389/fpls.2019.00405)
Supplement: Supplementary file 1 [file Table_1.pdf]

Supplementary Material

The Phosphorus Economy of Mediterranean Oak Saplings Under Global Change

Inga Dirks[1†], Julia Köhler[2], Shimon Rachmilevitch[1], Ina C. Meier [2*]

[1]French Associates Institute for Agriculture and Biotechnology of Drylands, Ben Gurion University of the Negev, Sede-Boqer Campus, Midreshet Ben Gurion 84990, Israel

[2]Plant Ecology, Albrecht-von-Haller Institute for Plant Sciences, Georg August University of Goettingen, 37075 Göttingen, Germany

†Present address: Department of Crop Sciences, Division Agronomy / Crop Science, Georg August University of Goettingen, 37075 Göttingen, Germany

*** Correspondence:**
Ina C. Meier
imeier1@uni-goettingen.de

1 Supplementary Figures and Tables

1.1 Supplementary Figures

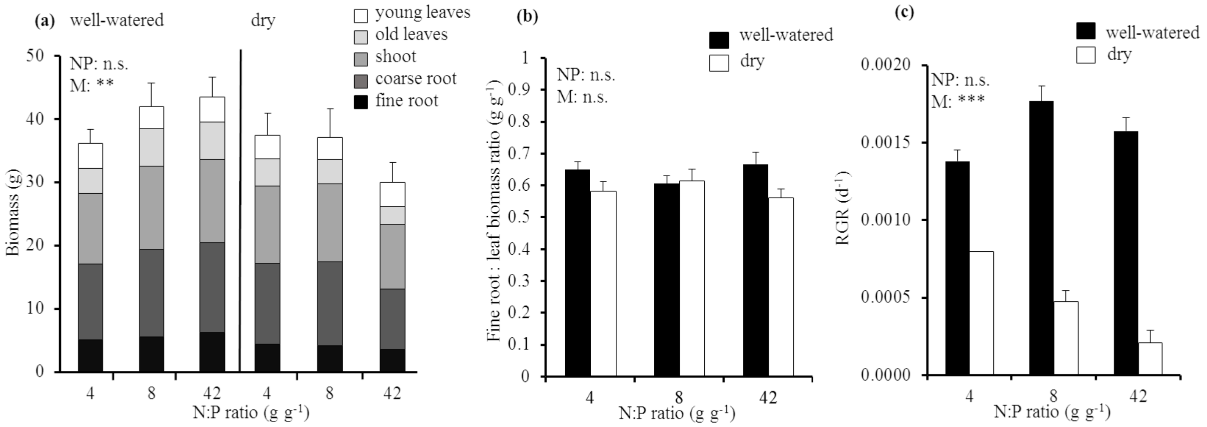

SUPPLEMENTARY FIGURE 1 (A) Biomass, **(B)** fine root:leaf biomass ratio, and **(C)** relative growth rate (RGR) of young Palestine oak (*Quercus calliprinos*) trees grown at increasing soil N:P ratio and decreasing soil moisture in a climate chamber. Shown are means and standard errors for replicate saplings (n = 48). The results of two-way ANOVAs on the significance of the effect of soil N:P ratio (NP) or soil moisture (M) are indicated by asterisks (*** and ** for $p \leq 0.001$ and 0.01; n.s., not significant).

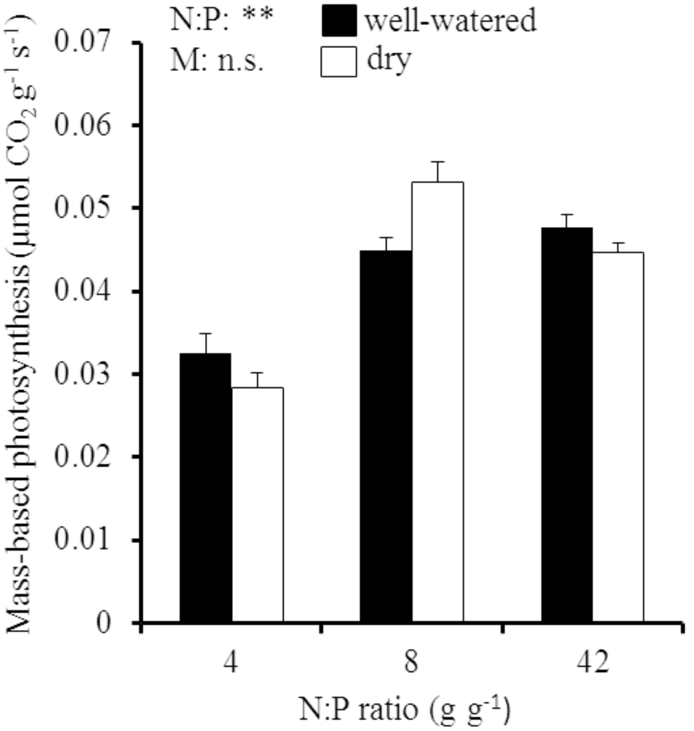

SUPPLEMENTARY FIGURE 2 Mass-based instantaneous late-summer photosynthesis of young Palestine oak (*Quercus calliprinos*) trees grown at increasing soil N:P ratio and decreasing soil moisture in a climate chamber. Shown are means and standard errors for replicate saplings (n = 24). The results of two-way ANOVAs on the significance of the effect of soil N:P ratio (NP) or soil moisture (M) are indicated by asterisks (** for $p \leq 0.01$; n.s., not significant).

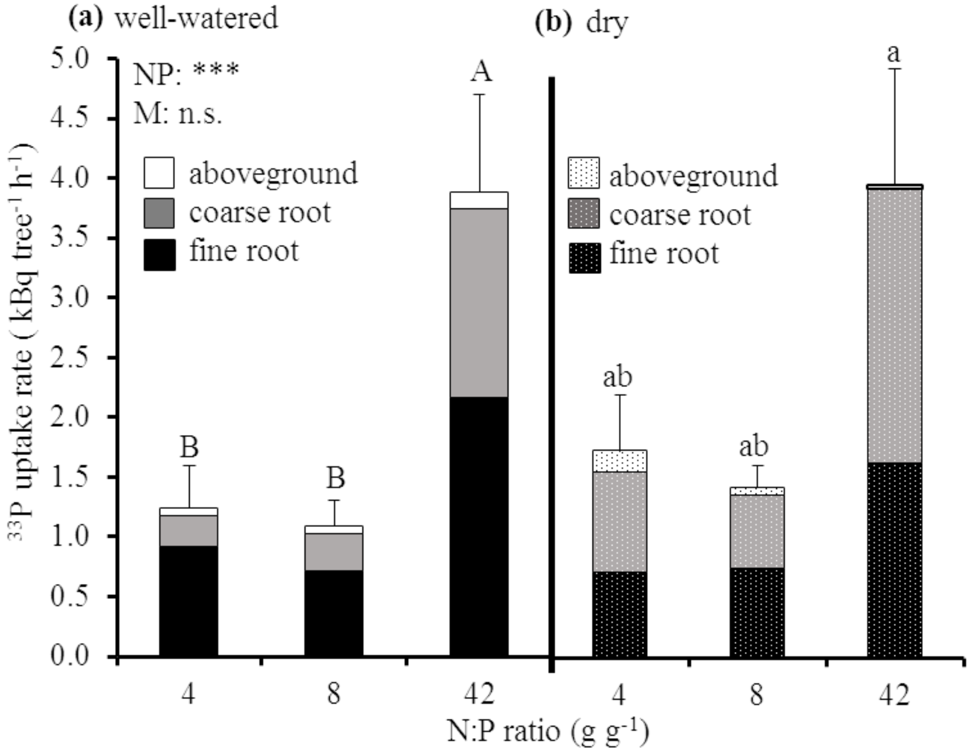

SUPPLEMENTARY FIGURE 3 ^{33}P uptake rate of fine roots, coarse roots and aboveground compartments of young Palestine oak (*Quercus calliprinos*) trees grown at increasing soil N:P ratio in **(A)** well-watered and **(B)** dry soil conditions in a climate chamber. Shown are means and standard errors for replicate saplings (n = 24). The results of two-way ANOVAs on the significance of the effect of the N:P ratio (NP) or soil moisture (M) are indicated by asterisks (*** for $p \leq 0.001$; n.s., not significant). Significant differences at $p \leq 0.05$ between soil N:P ratios are indicated for well-watered soil by different upper-case letters and for dry soil by different lower-case letters.

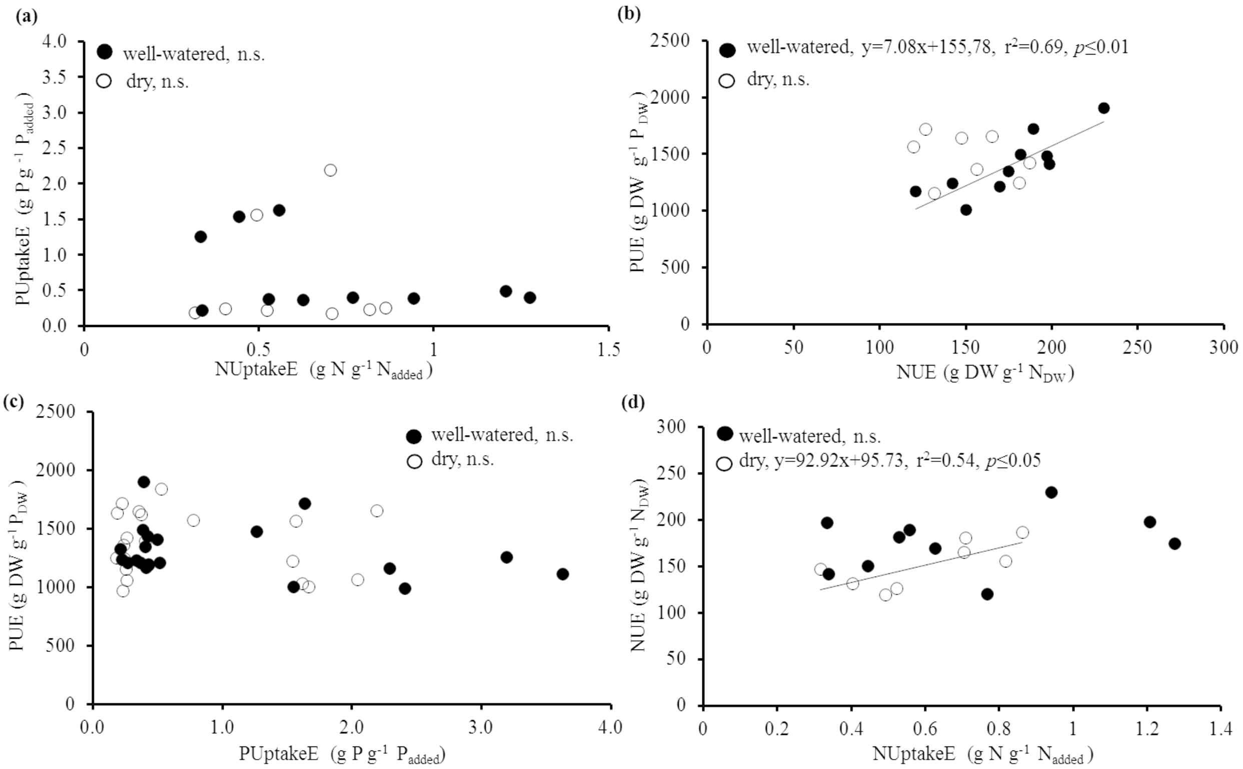

SUPPLEMENTARY FIGURE 4 Regression analyses on the dependence of **(A)** long-term P uptake efficiency (PUptakeE) from N uptake efficiency (NUptakeE) **(B)** P use efficiency (PUE) from N use efficiency (NUE), **(C)** PUE from PUptakeE, and **(D)** NUE from NUptakeE in young Palestine oak (*Quercus calliprinos*) trees grown at increasing soil N:P ratio and decreasing soil moisture. Significant relations are indicated for well-watered soil by black lines and for dry soil by grey lines ($p \leq 0.05$).

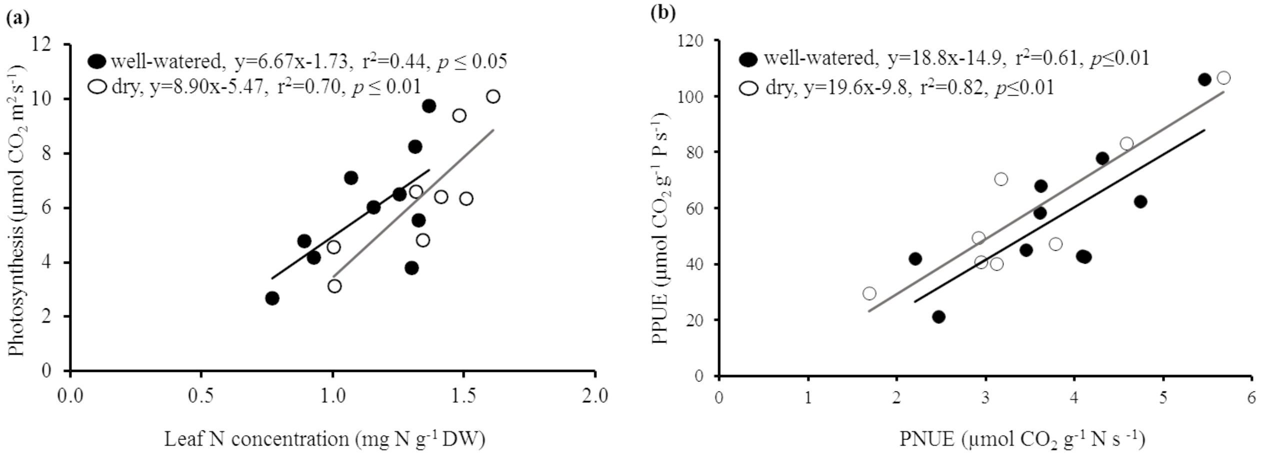

SUPPLEMENTARY FIGURE 5 Regression analyses on the dependence of **(A)** area-based instantaneous late-summer photosynthesis from leaf N concentration and **(B)** instantaneous late-summer photosynthetic P use efficiency (PPUE) from instantaneous late-summer photosynthetic N use efficiency (PNUE) in young Palestine oak (*Quercus calliprinos*) trees grown at increasing soil N:P ratio and decreasing soil moisture. Significant relations are indicated for well-watered soil by black lines and for dry soil by grey lines ($p \leq 0.05$).

1.2 Supplementary Table

SUPPLEMENTARY TABLE 1 Two-factorial analyses of variance (ANOVA) on the significance of the effects of the soil N:P ratio (NP), soil moisture (M), and their interaction on the variance of fine root or young leaf P concentrations, fine root or young leaf N:P ratio, area-based instantaneous late-summer photosynthesis (A_{800}), and specific leaf area (SLA) of young Palestine oak (*Quercus calliprinos*) trees. Given are *F* values and the probabilities of error *p*. Response variables were log-transformed to resemble normality. Significant effects ($p \leq 0.05$) are indicated by bold letters (n = 48 for P concentrations and SLA; n = 24 for N:P ratios).

	$P_{Fine\ roots}$		$P_{Young\ leaves}$		$N:P_{Fine\ roots}$		$N:P_{Young\ leaves}$		A_{800}		SLA	
	F	***p***	***F***	***p***	***F***	***p***	***F***	***p***	***F***	***p***	***F***	***p***
NP	2.32	0.11	2.31	0.11	0.23	0.80	**5.38**	**0.02**	**8.02**	**0.001**	**3.50**	**0.04**
M	0.00	0.95	0.23	0.63	4.09	0.07	2.78	0.12	0.02	0.89	1.05	0.31
NP*M	1.19	0.31	0.83	0.45	0.18	0.83	2.34	0.14	0.44	0.65	2.25	0.12
